# Overexpression of *SEPALLATA3*-like Gene *SnMADS37* Generates Green Petal-Tip Flowers in *Solanum nigrum*

**DOI:** 10.3390/plants14131891

**Published:** 2025-06-20

**Authors:** Siming Yuan, Chun-Lan Piao, Xinyu Zhang, Min-Long Cui

**Affiliations:** Key Laboratory of Quality and Safety Control for Subtropical Fruit and Vegetable, Ministry of Agriculture and Rural Affairs, Collaborative Innovation Center for Efficient and Green Production of Agriculture in Mountainous Areas of Zhejiang Province, College of Horticulture Science, Zhejiang A&F University, Hangzhou 311300, China

**Keywords:** Solanaceae, *Solanum nigrum*, MADS-box, *SEPALLATA3*-like gene *SnMADS37*, petal development, overexpression, chlorophyll accumulation, green petal-tip flower

## Abstract

The *SEPALLATA3* (*SEP3*)-like MADS-box genes play crucial roles in determining petal identity and development in the petunia and tomato of Solanaceae. *Solanum nigrum* is a self-pollinating plant in the Solanaceae family, and produces white flowers. However, the mechanisms controlling the transition from green to white petals during flower development remain poorly understood. In this study, we isolated a flower-specific *SEP3*-like gene, *SnMADS37*, from *S. nigrum*, and investigated its potential role in chlorophyll metabolism during petal development. Our results show that quantitative RT-PCR analysis demonstrates that *SnMADS37* is exclusively expressed in petals and stamens during early floral bud development. Overexpression of *SnMADS37* clearly enhanced the number of petals, promoting the formation of additional petal-like tissues in stamens and extra organs in some fruits. Moreover, fully opened transformed petals exhibited notable chlorophyll accumulation at their tips and veins, whereas silencing of *Snmads37* clearly inhibited petal expansion and reduced green pigmentation in early flower buds. Additionally, the transformed green petals exhibited distinct conical epidermal cells in the green regions, similar to wild type (WT) petals. Our results demonstrate that *SnMADS37* plays a critical role in regulating petal identity, expansion, and chlorophyll metabolism during petal development. These findings provide new insights into the functional diversification of *SEP3*-like MADS-box genes in angiosperms.

## 1. Introduction

Flower color is an important trait in ornamental plants. *Solanum nigrum* is a member of the economically significant Solanaceae family with the eggplant, potato, and petunia [1]. It is globally important for agriculture, human health, and biotechnology [2,3,4]. The flower of *S. nigrum* exhibits a typical zygomorphic structure, consisting of five sepals, five fused white petals, five stamens, and a fused carpel. Notably, the petals initially contain chlorophyll, which diminishes as the flower matures, resulting in a transition to a white appearance.

MADS-box proteins are a class of transcription factors found in a wide range of organisms, from yeast to humans [5,6]. In plants, the MADS-box genes influence various aspects of development, including root [7], fiber [8], floral organ [9], and fruit development [10,11]. Furthermore, molecular genetic studies of the model plants such *Arabidopsis*, *Antirrhinum*, and petunia have demonstrated that MADS-box transcription factors play a crucial role in determining floral organ identity and are codified in the ABCE model, which postulates four gene functions, A, B, C, and E, that act in overlapping concentric domains of the meristem to specify the floral organs. For instance, A + E specifies sepal, A + B + E specifies petals, B + C + E specifies stamens, and C + E specifies carpel identity in the flowers [12,13,14,15].

*SEPALLATA (SEP)* is an E-class MADS-box gene that acts as an important mediator in various aspects of flower development, working in conjunction with A-, B-, and C-class MADS-box genes. The *SEP*-like genes have been isolated from numerous plant species, including *Arabidopsis*, petunia, and tomato [13,16,17,18]. In *Arabidopsis*, four *SEP* family genes, i.e., *AtSEP1*, *AtSEP2*, *AtSEP3*, and *AtSEP4*, exhibit slightly different expression patterns but are all essential for the specification of floral organs [13,19]. The *Atsep3* single mutant displays only subtle phenotypic changes; however, the *Atsep1/Atsep2/Atsep3* triple mutants exhibit an abnormal phenotype in which the inner three whorls of floral organs are converted into sepals. In the *Atsep1/Atsep2/Atsep3/Atsep4* quadruple mutants, all the floral organs are converted into leaf-like structures [13,20]. In petunias, an *AtSEP3* ortholog *fbp2* mutant exhibit greenish petals and ectopic inflorescences originating from the third floral whorl [18]. In *Gerbera* of Asteraceae, the *SEP3* gene *GRCD5* (*GERBERA REGULATOR OF CAPITULUM DEVELOPNT 5*) shows unique functions in petal development. However, *GRCD1/2* belongs to the *SEP3* clade, and appears to have sub-functionalized to regulate stamen and carpel identity [21,22,23]. In contrast, overexpression of *AtSEP3* induces early flowering and activates the *AtAPETALA3* (*AtAP3*) and *AtAGAMOUS* (*AtAG*) genes [24]. Similarly, the ectopic expression of a *LaSEP3*-like gene in lavender results in early flowering in *Arabidopsis* [25]. Unlike, the ectopic expression of a wheat *SEP3*-like gene, *TaMADS1* leads to early flowering, alters floral organ development including the conversion of sepals into leaf-like structures, and reduces the numbers of petals and stamens in transformed *Arabidopsis* [26]. Furthermore, the ectopic expression of a homolog of *AtSEP3, BpMADS1,* decreases the number of floral organs or whole whorls along with petaloid or carpelloid sepals in *Arabidopsis* [27]. Therefore, *SEP* genes play an important role in the activities of A-, B-, and C-function genes in the development of petals, stamens, and carpels in flowering plants.

In this study, we isolated the *SnMADS37* gene and reported on its potential roles in *S. nigrum*. Our results indicate that the expression of *SnMADS37* is restricted to the petals and stamens. Overexpression of this gene increased the number of petals and led to additional organ development in both the stamens and fruits. Furthermore, it influenced chlorophyll accumulation in the petals, suggesting that *SnMADS37* plays an important role in the regulation of petal development, resulting in green petal-tip flowers in transformed *S. nigrum.*

## 2. Results

### 2.1. Isolation of SEPALLATA3-like Gene SnMADS37

**Figure 1:**
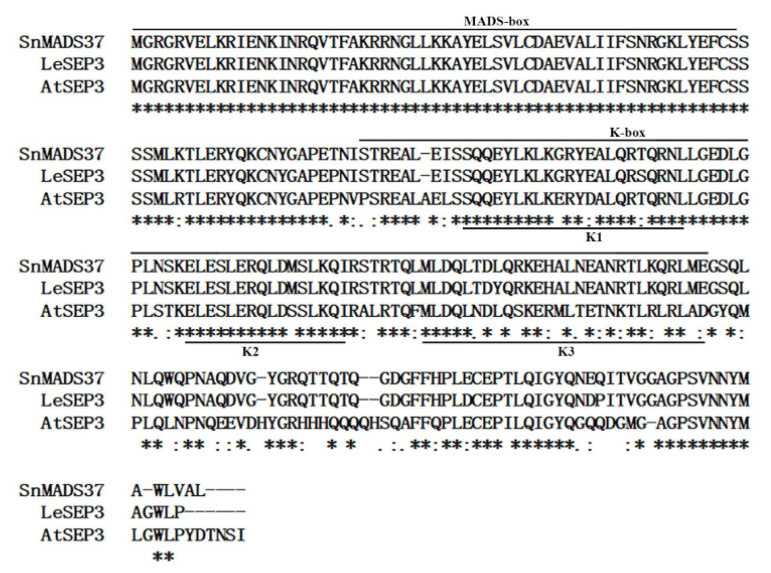
**Sequence comparison between *SnMADS37* and related MADS-box proteins.** The deduced amino acid sequence aligned with the sequences of AtSEP3 *(Arabidopsis*) and LeSEP3 (tomato). The MADS-box and K-box domains are indicated by lines above, and the three sub-domains in the K-box, namely K1, K2, and K3, are underlined.

Based on bioinformatics analysis of the transcriptome data of *S. nigrum* and BLAST searches (www.ncbi.nlm.nih.gov), two out of 88 MADS-box genes were found to be closely related to the *AtSEP3* in *Arabidopsis*. Furthermore, a comparison of the amino acid sequences of AtSEP3 in *Arabidopsis* and LeSEP3 in tomato revealed a candidate gene, *SnMADS37*. The full amino acid sequence of SnMADS37 showed similarities of 63.7% with AtSEP3 and 98.3% with LeSEP3, respectively (Figure 1). This led to the identification of *SnMADS37* as an *SEP3*-like gene in *S. nigrum*. To confirm the sequence of *SnMADS37,* the first strand cDNA from the inflorescence sample was amplified with the set of gene-specific primers SnMADS37-F2/SnMADS37-R2 (Table S1). The resulting 729 bp fragment encoded 242 amino acids, which corresponded to the primary transcriptome data (Figure 1).

### 2.2. Phylogenetic Tree Analysis

**Figure 2:**
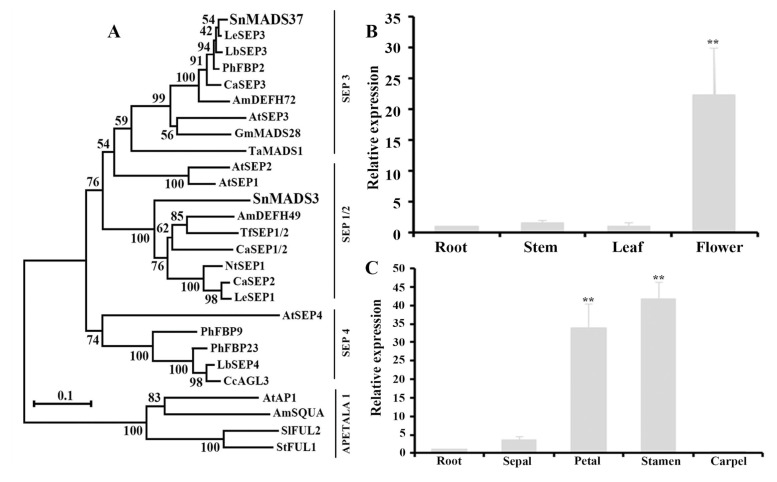
**Phylogenetic analysis and quantitative RT-PCR analysis of *SnMADS37*.** (**A**) *SnMADS37* identified in this study with selected *SEP*-like genes. The tree was constructed using the neighbor-joining method using MEGA 11 software and the bootstrap values for 1000 replicates. (**B**) Relative expression analysis of *SnMADS37* in the roots, leaves, stem, and early small inflorescence (Inflo); (**C**) Relative expression analysis of *SnMADS37* in the sepal, petal, stamen, and carpel of earlier stage 1 in supplemental Figure S2. Asterisks indicate statistically significant differences (*n* = 3; ** *p* < 0.01).

The alignment of the *SnMADS37* protein sequence with the protein sequences of known MADS-box genes from various plant species revealed highly conserved MADS-box and K-box domains (Figure 1). The deduced amino acid sequence of *SnMADS37* was 100% identical to both that of *LeSEP3* from tomato and that of *AtSEP3* from *Arabidopsis* in the MADS-box DNA-binding domain (Figure 1). The complete protein sequence of SnMADS37 exhibited 98.3% and 63.7% identity with LeSEP3 from tomato and AtSEP3 from *Arabidopsis*, respectively. Additionally, phylogenetic tree analysis demonstrated a homologous evolutionary relationship between SnMADS37 and SEP3 proteins from other plants. *SnMADS37* was closely related to *LeSEP3* from tomato and *PhFBP2* from petunia within the same Solanaceae family, while SnMADS3 exhibited a highly conserved MADS-box domain and full sequence similarity with both *SnMADS37* and *AtSEP3* (Figure S1); phylogenetic analysis places it within the SEP1 subfamily (Figure 2A). Therefore, sequence analysis suggests that *SnMADS37* is an *SEP3*-like MADS-box transcription factor in *S. nigrum*.

### 2.3. Expression Analysis of SnMADS37

To investigate the expression of *SnMADS37* in WT *S. nigrum* plants, quantitative RT-PCR analysis was performed using total RNA isolated from roots, mature leaves, stems, and early small inflorescences (Figure 2B). A relatively strong expression of *SnMADS37* was observed in the inflorescence; in contrast, its expression greatly reduced in the stems and leaves (Figure 2B). Additionally, strong expression of *SnMADS37* was detected in the petals and stamens; however, its expression in the sepals and carpels of early stage 1 floral buds was remarkably decreased (Figure 2C and Figure S1). These results suggest that *SnMADS37* is likely involved in the development of petals and stamens in *S. nigrum*.

**Figure 3:**
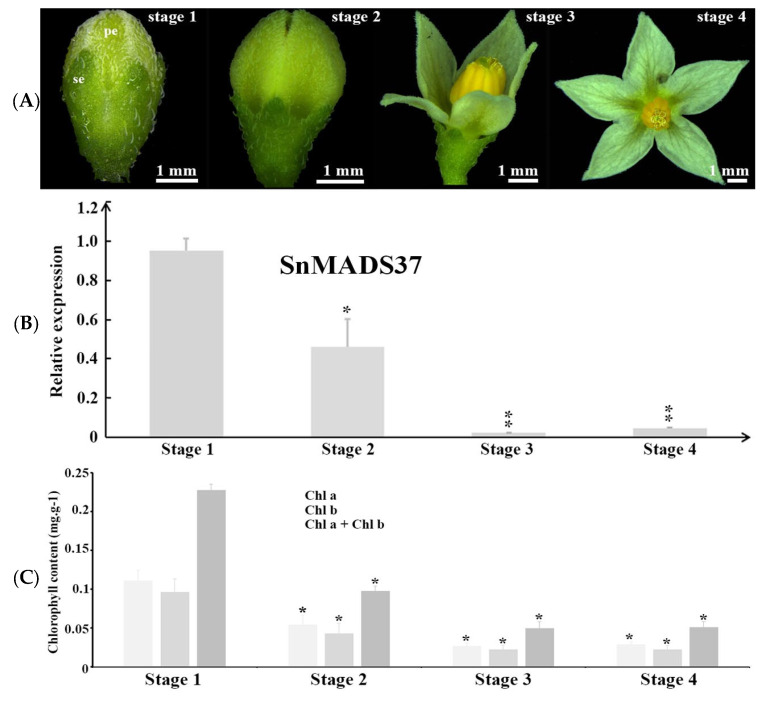
**Quantitative RT-PCR analysis of *SnMADS37* in four developmental stages of wild type (WT) flowers**. (**A**) Comparison of the shapes and chlorophyll colorations of the petals among four flower developmental stages. se: sepal; pe: petal. (**B**) Analysis of the relative expression of *SnMADS37* in four flower developmental stages. (**C**) Chlorophyll content comparison among four flower developmental stages. Asterisks indicate statistically significant differences (*n* = 3; * *p* < 0.05; ** *p* < 0.01).

Further, to investigate the relationship between green color and *SnMADS37* expression during petal development, we performed qRT–PCR analysis at four stages of flower development (Figure 3A): stage 1, the earliest visible floral buds with small green petals; stage 2, elongated petals with an unopened flower; stage 3, initiation of flower opening; and stage 4, fully opened flower. The relative expressions of the B-function genes *SnGlobosa* (*SnGLO*) and *SnDeficiens* (*SnDEF*) exhibited similar patterns across all stages (Figure S2). However, strong expression of *SnMADS37* was detected in stage 1, while its relative expression gradually decreased in stage 2. In stages 3 and 4, the expression of *SnMADS37* was significantly suppressed (Figure 3B). We further investigated the chlorophyll content in the four developmental stages (Figure 3C) and identified about 0.23 mg∙g^−1^ chlorophyll in stage 1 petals. During the other three stages, the chlorophyll content gradually decreased, becoming 0.098, 0.045, and 0.051 mg∙g^−1^, respectively. In addition, the expression of *SnMADS37* matched the chlorophyll decrease during four stages. These results suggest that *SnMADS37* may be involved in the chlorophyll metabolism during petal development.

### 2.4. Overexpression of SnMADS37 Leads to Morphological Changes in the Flower

**Figure 4:**
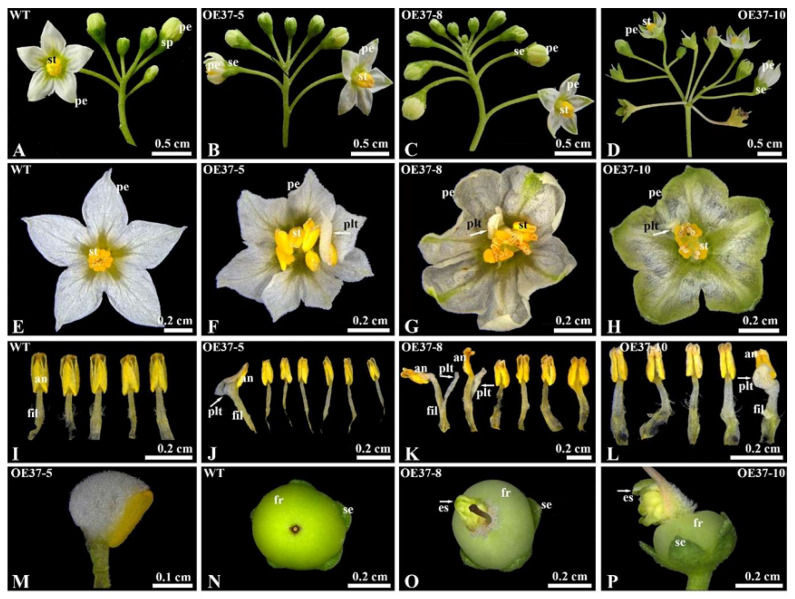
Flower phenotype comparison between WT and transformed *S. nigrum* plants grown in a climate chamber (25 °C, 16 h light condition). (**A**) Inflorescence of WT; (**B**) inflorescence of OE37-5; (**C**) inflorescence of OE37-8; (**D**) inflorescence of OE37-10; (**E**) flower of WT, showing five petals and stamens; (**F**) flower, showing seven petals and stamens; (**G**) flower, showing six petals and stamens; (**H**) flower, showing notable greenish petals; (**I**) five stamens of WT plant from panel (**E**); (**J**) seven stamens of the case in panel (**F**); (**K**) six stamens of the case in panel (**G**); (**L**) five stamens of the case in panel (**H**); (**M**) magnified petaloid anther of the case in panel (**F**); (**N**) WT fruit; (**O**) Extra organ developing transformed fruits of OE37-8; (**P**) Extra organ developing transformed fruits of OE37-10. The increased petal-like tissue in panels (**F**–**H**,**J**,**K**), and (**L**) formed an additional organ on the fruit in panels (**O**,**P**), which is indicated by the white arrow (se: sepal; pe: petal; st: stamen; fil: filament; an: anther; fr: fruit; plt: petal-like tissue, and es: extra shoot like organ).

To investigate the function of *SnMADS37*, it was ectopically expressed in *S. nigrum*, driven by the CaMV 35S promoter. Twelve overexpressing transgenic plants were selected following antibiotic selection (kanamycin) and PCR analysis. Among these, four independent transgenic lines with clear green petals were used for further examination. The transgenic plants exhibited complex phenotypic alterations in their flowers (Figure 4). The inflorescences and collations of early floral buds in most transgenic plants did not display visible differences compared to those of WT plants (Figure 4A–C). However, some flowers in the OE37-10 line developed into secondary inflorescences (Figure 4D). The sepals of the transgenic flowers closely resembled those of the WT flowers, with no obvious alterations in their number or shape observed (Figure 4A–D). Nevertheless, the fully opened petals exhibited greenish tips and vents (Figure 4B–D,F–H). Flowers with six to seven petals and stamens were present (Figure 4F,G,J,K), and additional petal-like tissues also developed on the stamens of the transformed flowers (Figure 4F–H,J–L). Furthermore, some fruits from the transgenic lines exhibited extra shoot like organ formation (Figure 4O,P), which was comparable to that of WT fruits (Figure 4N). Based on these results, *SnMADS37* is implicated in the regulation of flower organ identity and development, particularly in petal development.

### 2.5. SEM and Sliced Section Analysis

**Figure 5:**
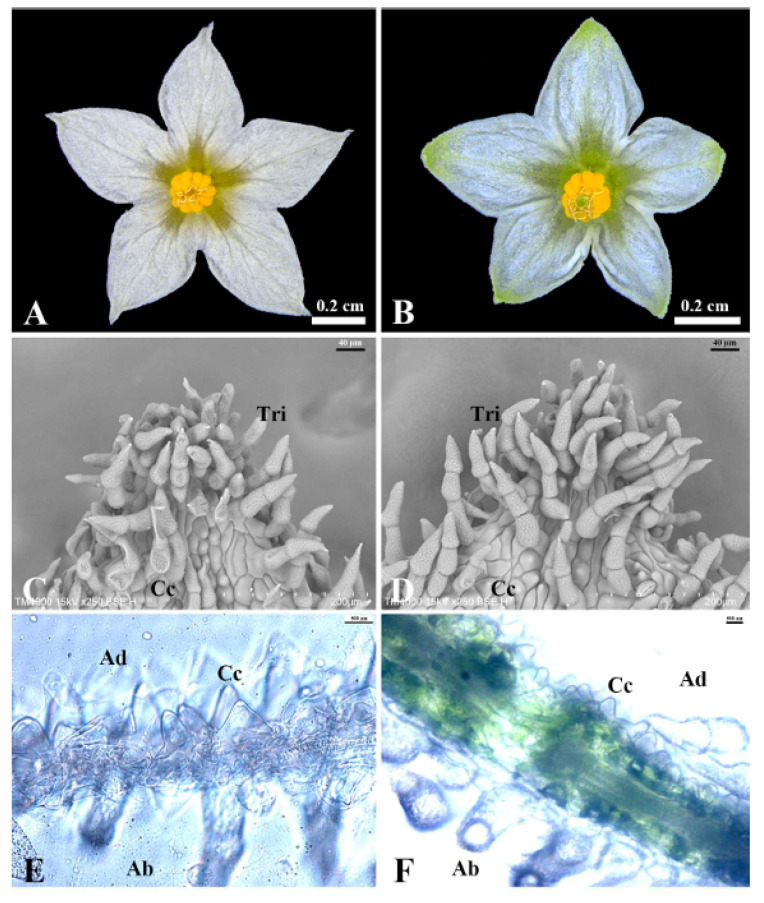
**Petal-tip comparison between the WT and OE37-10 flower.** (**A**) WT flower; (**B**) OE37-10 flower; (**C**) SEM image of adaxial surface of petal-tip in panel (**A**); (**D**) SEM image of adaxial surface of petal-tip in panel (**B**); (**E**) transection of petal-tip in panel (**A**); (**F**) transection of petal-tip in panel (**B**) (Ad: adaxial of petal; Ab: abaxial of petal; Tri: trichome; Cc: conical cell).

Similar to the WT petal, most of the region of the transformed petal appeared whitish; however, the tip and vein regions exhibited a greenish hue (Figure 4A–D and Figure 5A,B). To determine how *SnMADS37* influences petal-tip coloration and structure, we chose the remarkable OE37-10 line and a WT plant was investigated using SEM and sliced sections (Figure 5A,B). We observed distinct conical epidermal cells and trichomes on the adaxial surface of the petal-tip in the OE37-10 line, which resembled those of the WT petal-tip (Figure 5C,D). Furthermore, the cross-section of the petal-tip section in the OE37-10 line was thicker, with a noticeable accumulation of chlorophyll observed on the inner side of the petal (Figure 5E,F), in contrast to the thinner, whitish WT petal that lacked chlorophyll accumulation (Figure 5C,E).

### 2.6. Expression of SnMADS37 Affects Chlorophyll Content in Flower

**Figure 6:**
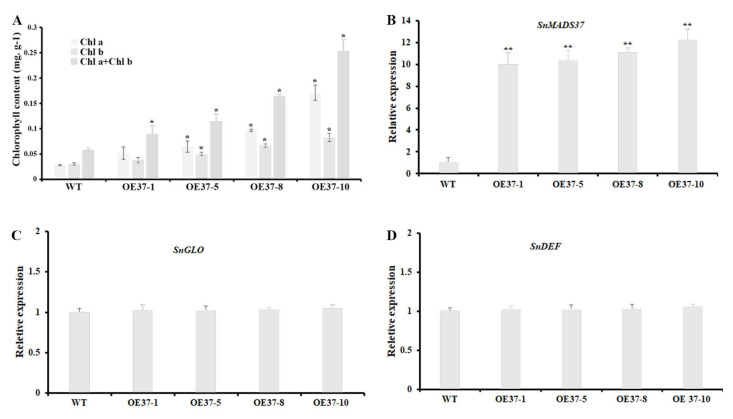
Comparison of chlorophyll content and relative expression analysis of B-function genes in fully opened stage 4 petals among a WT and four transgenic *S. nigrum* plants. (**A**) Analysis of the total chlorophyll content from petals of a WT and four transgenic plants; (**B**) *SnMADS37* expression analysis from petals of a WT and four transgenic plants; (**C**) B-function gene *SnGLO* expression analysis from petals of a WT and four transgenic plants; (**D**) B-function gene *SnDEF* expression analysis from petals of a WT and four transgenic plants. OE37-1, 5, 8, and 10: four transformed plants with pBI-*35S::SnMADS37*. Asterisks indicate statistically significant differences between the WT and transgenic lines (*n* = 3; * *p* < 0.05; ** *p* < 0.01). Chl a: chlorophyll a; Chl b: chlorophyll b.

To determine whether the expression of *SnMADS37* affects chlorophyll content in flowers, we investigated the total chlorophyll content in the petals of the WT and four transformed plants using a spectrophotometer. Chlorophyll was detected in the petals of all transformed plants, with its content being approximately 1.5- to 4.4-fold higher than that in the WT petals. This chlorophyll content and coloration corresponded with the expression of *SnMADS37* in WT and transgenic petals (Figure 6A,B). However, no significant differences in the expression of B-function genes *SnGLO* and *SnDEF* were detected in the petal of WT and transgenic lines (Figure 6C,D). These results indicate that the expression of *SnMADS37* likely affects chlorophyll metabolism, resulting in chlorophyll accumulation in the transformed petals.

**Figure 7:**
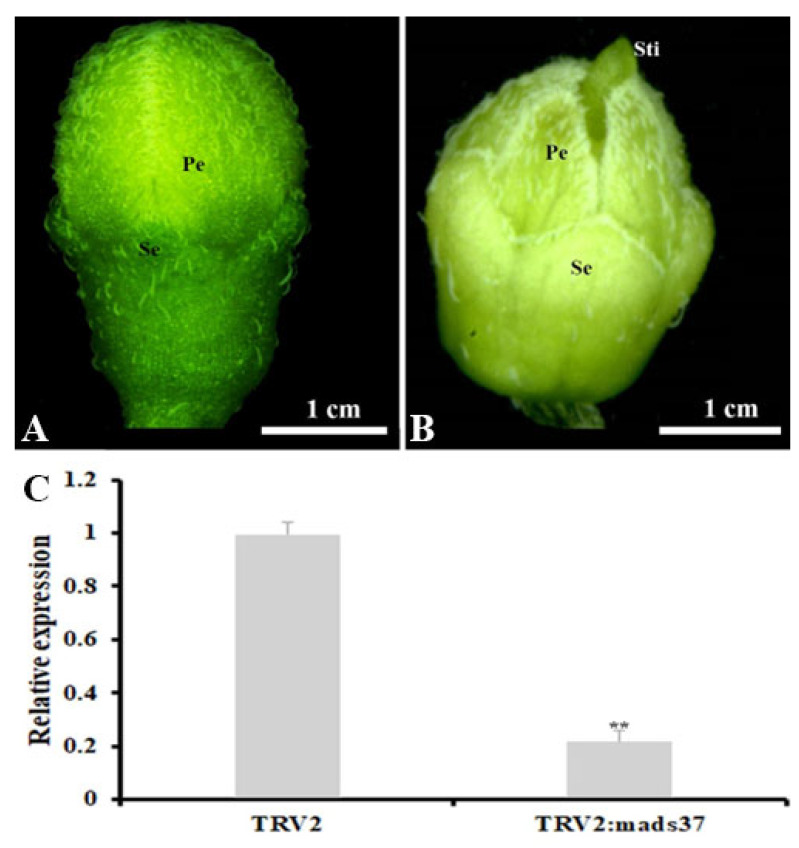
**Comparison of phenotype between a control and an *SnMADS37*-silenced stage 1 floral bud of *S. nigrum* by VIGS treatment.** (**A**) a control stage 1 floral bud treated with GV3101/*TRV2;* (**B**) an *Snmads37*-silenced stage 1 floral bud treated with GV3101/*TRV2::Snmads37*; (**C**) total RNA was extracted from stage 1 floral bud in (**A**) and (**B**), respectively, and relative expression of *SnMADS37* was detected by qRT-PCR analysis. Asterisks indicate statistically significant differences (*n* = 3; ** *p* < 0.01). Se, sepal; Pe. petal; Sti, stigma.

To confirm our hypothesis, we employed the virus-induced gene silencing (VIGS) method to investigate the potential role of *SnMADS37* in flower development. A 260-bp fragment from the 3′ end of *SnMADS37* was used to construct the VIGS vector pTRV2::SnMADS37, with the empty pTRV2 vector as a control. These constructs were infiltrated into the apical tips of 5-week-old *S. nigrum* plants. Subsequently, early altered floral buds were observed in 4 out of 12 treated plants after 2 weeks of culture in a growth chamber at 25 °C under 16/8 h of light/dark conditions (Figure 7). We found that the control flower bud treated with GV3101/*TRV2* showed green sepals and closed greenish petals, which were similar to wild type flower buds (Figure 7A and Figure S1). Compared to the GV3101/*TRV2* control flower bud, some flower buds treated with GV3101/*TRV2::mads37* displayed clear morphological changes, such as shrunk and reduced green color petals, and wide and pale green sepals and some floral buds displayed distinct stigma; we also observed that the relative expression of *SnMADS37* was greatly downregulated in the *Snmads37*-silenced floral buds compared to the control floral buds (Figure 7B,C). Furthermore, we also found likely reduced relative expression of a chlorophyll-related biosynthetic gene *SnCHLH* (Mg-chelatase subunit H gene) and two chlorophyll-related degradation genes *SnCLH* (hydroxymethyl chlorophyll a reductase) and *SnPPH* (pheophytinase) by qRT-PCR analysis (Figure S3). These results suggest that *SnMADS37* is not only involved in the regulation of petal expansion but also negatively affects chlorophyll degradation during the flower development; the green petal-tip flower may be caused by *SnMADS37* expression.

## 3. Discussion

Flower morphogenesis is a highly robust and standardized developmental process. In the past three decades, significant transcriptional regulators that govern floral development have been identified in model organisms such as *Antirrhinum* and *Arabidopsis* [9,12]. The “floral quartet model” posits that floral homeotic proteins form organ-specific tetrameric complexes, with their interactions being facilitated by functionally redundant SEP proteins (SEP1–SEP4) in *Arabidopsis*. Within this model, a quartet complex consisting of APETALA1, APETALA3, PISTILLATA, and SEP is responsible for specifying petal identity. Additionally, previous research has also demonstrated that *SEP3* family genes are involved in petal development regulation in the tomato and petunia of Solanaceae [18,28]. In this study, we isolated and characterized the MADS-box gene SnMADS37 from S. nigrum. Phylogenetic analysis of SnMADS37 within a clade with LeSEP3 from tomato [28], PhFBP2 from petunia [18], and AtSEP3 from Arabidopsis [13] (Figure 2A) was conducted. Moreover, the deduced amino acid sequence of SnMADS37 contains the highly conserved MADS-box and K-box domains, similar to LeSEP3 of the tomato (Figure 1). These structural similarities in the amino acid sequence and phylogenetic analysis suggest that *SnMADS37* may function as an *SEP3*-like MADS-box gene in *S. nigrum*.

Members of the MADS-box transcription factor play an essential role in regulating floral organ identity, which is specified by the combined protein interactions of ABCE-class MADS-box domain transcription factors. The E-class *SEP* transcription factors are particularly significant in the regulation of floral organ identity and development across various plants [9,12,17,29]. In this study, the overexpression of the *SEP3*-like gene *SnMADS37* resulted in various floral developmental alterations in transgenic plants, such as increased numbers of flowers (Figure 4C,D), the development of flowers with 6 to 7 petals and stamens (Figure 4F–H), and the occurrence of ectopic petal formation on staminal anthers. Furthermore, we observed the emergence of additional organs on early green fruits, while certain flowers appeared to revert to secondary inflorescences in transgenic *S. nigrum* (see Figure 4D). These findings imply that *SnMADS37* is critical for floral organ specification and petal development in *S. nigrum*. Similar functions of SEP3-like genes have been identified and characterized in transformed tobacco. For example, overexpression of the soybean *SEP3*-like gene *GmMADS28* in tobacco induces early flowering and alters the floral organs’ morphology, including stamens, sepals, and petals [30]. Similarly, *PlacSEP3* overexpression in transformed tobacco promotes early flowering and produces lateral branches [31]. In *Arabidopsis,* overexpression of the wheat *SEP3*-like gene *TaMADS1* leads to early flowering, converts the sepal into a leaf-like structure, and reduces petal and stamen numbers [26]. Moreover, loss of function of *FBP2* in petunias led to secondary inflorescence development in the third whorl [32,33], *TM5* downregulation in the tomato resulted in a loss of determinacy in the center of the flower [34], and overexpression of *JcSEP3* in *Jatropha curcas* induced male floral organ formation despite broad expression across floral tissues [35]. These results suggest that *SnMADS37*, probably an *SEP*-like gene, plays multifaceted roles in floral development, with phenotypic outcomes likely modulated by expression levels. However, since *S. nigrum* may harbor additional undetected *SEP*-like genes with redundant functions, which specific contributions it makes to floral organ identity and development requires further investigation.

In *S. nigrum,* the petals of closed flower buds are green (Figure 3A). Once the flower opens, the upper part of the petal expands, and chlorophyll degrades, resulting in whitish petals (Figure 3A). In this study, the petals of transformed *S. nigrum* with the 35S::SnMADS37 showed clear chlorophyll accumulation, displaying greenish tips and veins that were comparable to those of WT petals (Figure 5). The morphological changes are similar to the loss of function seen in the *SEP3* ortholog *FBP2* mutant in petunias [18]. However, while the petals of the *fbp2* mutant exhibit leaf or sepal-like characteristics, such as stomata in the greenish segments of petals, the petals of transformed *SnMADS37* showed conical epidermal cells on the adaxial side and lacked stomatal structures. This is indicative of petal identity in angiosperms [36], and the epidermal cell structure closely resembles that of WT petals (Figure 5A,B). Therefore, the chlorophyll accumulation observed in the petals of transformed *S. nigrum* is distinct from that in leaf-like flowers, resulting from loss-of-function mutations in SEP-like genes, such as *fbp2* in petunias [18] and the *sep1/sep2/sep3* triple mutant in *Arabidopsis* [19].

In addition, we observed that floral buds with reduced *SnMADS37* expression exhibited wider and pale green sepals, along with shriveled petals showing decreased green pigmentation, and the floral buds displayed a more prominent stigma compared to both the control pTRV2-treated and overexpression flower buds (Figure 5B–D and Figure 7), implying that *SnMADS37* is directly or indirectly involved in chlorophyll degradation during petal development. On the other hand, we focused solely on *SnMADS37*; however, other undetected *SEP*-like genes, such as *SnMADS3* (Figure 2A), may have redundant functions in regulating floral organ identity in *S. nigrum*. Thus, the role of *SnMADS37* in floral organ identity and petal maturation warrants further investigation through transcriptomic and metabolomic analyses, genome editing, and in situ techniques.

Previous studies have reported that the MADS-box gene *SOC1* is involved in regulating morphological development and contributes to chloroplast biogenesis and biosynthesis, resulting in green petal formation in transformed tobacco [37]. Furthermore, the overexpression of *BpMADS* from *Betula platyphylla* significantly enhances chloroplast division, and the rate of photosynthesis in transformed tobacco [38]. In this study, the B-function genes *SnGLO* and *SnDEF* were likely not involved in the accumulation of chlorophyll during petal development (Figure 3 and Figure 6C,D). Conversely, chlorophyll accumulation correlates with the expression of *SnMADS37* during the petal development stages of both WT and transformed petals (Figure 3 and Figure 7A,B). We also found reduced expression of certain *SnCLH* and *SnPPH* genes, which are involved in chlorophyll degradation in the petals of *SnMADS37* overexpressed lines (Figure S3). Additionally, *SnMADS37* alone could not stimulate chlorophyll accumulation in the roots of transformed lines (Figure S4). Moreover, compared to WT and control floral buds, the silenced *Snmads37* floral petals reduced green coloration (Figure 7 and Figure S1). These findings suggest that *SnMADS37* negatively influences chlorophyll degradation during the petal development process. Consequently, the reduced rate of chlorophyll degradation associated with the expression of *SnMADS37* likely contributes to the accumulation of chlorophyll in the green petal-tips of transformed *S. nigrum*.

In this study, we observed that the overexpression of *SnMADS37* affected floral organ development and influenced chlorophyll accumulation in the petals. This finding indicates that *SnMADS37* plays a crucial role in floral organ identity and the petal development process in *S. nigrum*. Clearly, elucidating the role of the *SnMADS37* gene will not only enhance our understanding of the biological functions of *SEF* family genes but also provide novel insights into the significance of *SnMADS37* in regulating petals and other floral organ developments.

## 4. Materials and Methods

### 4.1. Plant Material and Growth Conditions

*Solanum nigrum* plants were cultivated on solid Murashige and Skoog (MS) medium [39] at 25 °C under a 16/8 h of light/dark conditions in a tissue culture room at Zhejiang A&F University. Fully expanded young leaves were used for transformation and other experiments.

### 4.2. SnMADS37 Gene Cloning

Total RNA was isolated from 100 mg of floral bud powder of *S. nigrum* using the Eastep^®^ Super Total RNA Extraction Kit (Promega, Madison, WI, USA). First-strand complementary DNA (cDNA) was synthesized from 5 µg of total RNA using the *TransScript*^®^ II First-Strand cDNA Synthesis SuperMix (TransGen Biotech, Beijing, China). Cloning of full-length *SnMADS37* was performed using the *TransStart*^®^ *FastPfu* DNA Polymerase (TransGen Biotech) and a set of gene-specific primers SnMADS37-F2/SnMADS37-R2 (Table S1), which were designed based on MADS-box-like transcription factor-derived transcriptome data of *S. nigrum*. Polymerase chain reaction (PCR) was performed under the following conditions: preliminary denaturation at 97 °C for 3 min, followed by 30 cycles of denaturation at 95 °C for 50 s, annealing at 60 °C for 1 min, extension at 72 °C for 1 min, and a final extension at 72 °C for 10 min. The amplified fragment was cloned using the pEASY-Blunt simple vector (TransGen Biotech), dubbed pEASY-MADS37, and confirmed via sequencing. The amplified 729-bp full-length cDNA was dubbed *SnMADS37* (DDBJ: LC782345).

### 4.3. Phylogenetic Tree Construction

The amino acid sequence of *SnMADS37* was aligned with certain *SEPALLATA*-like MADS-box gene sequences. Neighbor-joining phylogenetic trees with 1000 bootstrap replicates were constructed using Clustal W version 2.0 [40] and MEGA version 11 [41]. The bars indicate an evolutionary distance of 0.1%. The GenBank accession numbers of these proteins are as follows: *Arabidopsis thaliana* AtAP1 (NP_177074), AtSEP1 AK118608), AtSEP2 (AY727601), AtSEP3 (AF015552), and AtSEP4 (NM126418); *Antirrhinum majus* AmSQUA (CAA45228), AmDEFH49 (ACAA64741), and AmDEFH72 (CAA64742); *Solanum lycopersicum* SlFUL2 (ART88618), LeSEP1 (NP_001233911), and LeSEP3 (NP_001234384); *Petunia hybrida* PhFBP2 (M91666), PhFBP9 (AAK21249), and PhFBP23 (AF335241); *Solanum tuberosum* StFUL1 (NP_001275142); *Coffea arabica* CaSEP1/2 (AHW58036) and CaSEP3 (AHW58034); *Lycium barbarum* LbSEPL3 (ADP09004); *Torenia fournieri* TfSEP1/2 (BAO74162); *Nicotiana tabacum* NtSEPL 1 (XP_016466966); *Capsicum annuum* CaSEP2 (KAF3682829); *Capsicum chinense* CcAGL3 (PHU23937); *Lycium barbarum* LbSEP4 (AJW87597); *Glycin max* GmMADS28 (AJ878424); and *Triticum aestivum* TaMADS1 (AF543316).

### 4.4. Transcriptional Analysis of SnMADS37

Total RNA was isolated from the roots, leaves, stems, and inflorescences as well as the sepals, petals, stamens, and carpels of wild type *S. nigrum* (Figure S1) using an Eastep^®^ Super Total RNA Extraction Kit (Promega, Madison, WI, USA). Reverse transcription from 2 µg of total RNA to cDNA was performed using *EasyScript*^®^ First-Strand cDNA Synthesis Super Mix (TransGen Biotech) according to the manufacturer’s instructions. Each reaction volume was 10 μL, and PCRs were conducted as follows: 95 °C for 30 s, followed by 30 cycles of 5 s at 95 °C and 30 s at 60 °C. The relative expression levels of the target genes were calculated using the 2^−ΔΔCt^ method [42]. SnActin (Table S1) was used as an internal control to normalize the transcription levels of the target genes.

### 4.5. Construction of the Overexpression Vector pBI-35S::SnMADS37

The full-length *SnMADS37* cDNA coding region was amplified from the pEASY-MADS37 plasmid using a *Bam*H I-*SnMADS37F* and *Sac* I-*SnMADS37R* gene-specific primer set (Table S1) in combination with *TransStart*^®^ *FastPfu* DNA Polymerase (TransGen Biotech). The resultant was sub-cloned into a pEASY-Blunt vector for confirmation via sequencing. Using *Bam*H I and *Sac* I restriction sites, *SnMADS37* substituted the *GUS* gene in the pBI121 vector to produce pBI-*35S::SnMADS37*, containing the full-length *SnMADS37* coding region between the *35S* promoter and CaMV terminator. The pBI-*35S::SnMADS37* expression vector was electroporated into *Agrobacterium* GV3101 cells [43].

### 4.6. Transformation of S. nigrum

Young leaves of *S. nigrum* were used for transformation. Transformation was performed according to the method of Piao et al. [44], albeit slightly modified. The *Agrobacterium* strain GV3101/pBI-*35S::SnMADS37* was grown in 5 mL of liquid LB medium containing 50 mg∙L^−1^ kanamycin at 28 °C, shaken at 200 rpm for 24 h, and subsequently 30-fold diluted with liquid MS medium for inoculation. After four weeks of infection, the obtained adventitious shoots were transferred to a fresh solid MS medium containing 250 mg∙L^−1^ cefotaxime and 50 mg∙L^−1^ kanamycin, and transformed positive shoots were selected for PCR analysis. The selected transformed plants were maintained at 25 °C under a 16/8 h light/dark photoperiod condition in an artificial climate chamber and used in the subsequent experiments.

### 4.7. SnMADS37 Expression Analysis in the Transgenic S. nigrum

Total RNA was extracted from 100 mg of fresh young wild type (WT) leaves and five independent T_1_ transgenic *S. nigrum* plants, and first-strand cDNA synthesis was performed as described above. Semi-quantitative reverse transcription polymerase chain reaction (RT-PCR) analysis was performed using 20 ng first cDNA as a template and the set of gene-specific primers, namely SnMADS37-F2/SnMADS37-R2, and Actin gene of *S. nigrum* as a positive control (Table S1).

### 4.8. VIGS-Mediated Silencing of SnMADS37 in Solanum nigrum Flowers

To construct the SnMADS37 VIGS vector, we designed a primer set targeting a 260-bp fragment at the 3′ end of the gene, a region exhibiting low sequence identity with the *SnMADS3* gene. Using pTRV2 [45] as the backbone, we generated the VIGS vector pTRV2::SnMADS37. Both this construct and the helper vector pTRV1 were introduced into *A. tumefaciens* strain GV3101 via electroporation.

The silencing of *SnMADS37* in the flower of *S. nigrum* was performed with a slightly modified procedure described by Hartl et al. [46]. The *A.* strains GV3101/TRV1, GV3101/TRV2, and GV3101/TRV2::SnMADS37 were cultured in 100 mL LB medium supplemented with 50 mg·L^−1^ kanamycin and 25 mg·L^−1^ Rifampicin at 28 °C with shaking 200 rpm for 24 h. Cells were then harvested by centrifugation at 5000 rpm for 10 min, and the pellet was resuspended in 100 mL infiltration medium (1% sucrose, 10 mM MgCl_2_, 10 mM MES, 200 µM acetosyringone). After 3 h of incubation at 28 °C and 200 rpm, the GV3101/TRV1 suspension was mixed in equal volumes with either GV3101/TRV2::SnMADS37 or GV3101/TRV2 (control). The mixtures were diluted with infiltration medium to an OD_600_ of 0.3 for infiltration. For each treatment, six 5-week-old plants were used. The apical meristems were vacuum-infiltrated for a pressure of 60–70 mbar and 1 min slowly. The vacuum infiltration experiment was performed twice, and phenotypic observations were performed 10 days post-infiltration.

### 4.9. Scanning Electron Microscopy (SEM) Observations

Petal tissue samples of both wild type and T_1_ transgenic *SnMADS37-10* were prepared for SEM as previously described by Krizek [47]. SEM analyses were performed using a TM4000 instrument (Hitachi, Chiyoda City, Japan).

### 4.10. Slice Analysis of Petals

Fresh WT and T_1_ transgenic *SnMADS37-10* petal-tips were sliced into 40 μm thick sections using a Leica VT1000 slicer. The sections were covered with a cover glass for conducting microscopic observations. Images were captured using a Leica light microscope DM2500. Digital image processing was performed using Adobe Photoshop 7.0.

### 4.11. Measurement of Chlorophyll Pigment Content

The chlorophyll a and b contents were determined using the method of Zhou et al. [48], albeit slightly modified. Briefly, 50 mg of excised leaf disks or fully opened petals of wild type or T_1_ transgenic *S. nigrum* were fully submerged in 10 mL acetone:ethanol (2:1, *v*/*v*) buffered at 4 °C, and were left in the dark overnight to extract the chlorophyll pigments. The absorbances at 663 and 645 nm were measured for the chlorophyll extract, and the chlorophyll a and b levels were calculated following Zhou et al. [47]. Each sample was replicated three times, and statistical analyses were performed using SPSS 25.0.

## Data Availability

All data generated or analyzed during this study are included in the article and its Supplementary Materials.

## Supplementary Materials

The following supporting information can be downloaded at https://www.mdpi.com/article/10.3390/plants14131891/s1, Table S1: the primers used for *SnMADS37* gene cloning, detection of transformed plants, and RT-PCR analysis in this study; Figure S1: the green color of early wild type stage 1 floral bud (**A**), and with stamens (**B**) that removed the sepals and petals in Figure 4A, Figure S2: expression analysis of B-function gene in petal development stage of WT, Figure S3: relative expression analysis of chlorophyll-related genes in fully opened stage 4 petals among a WT and four transgenic *S. nigrum* plants, Figure S4: comparison of root coloration between a WT and five overexpressed SnMASD37 plants on MS medium + 200 mg/L cefotaxime after three weeks culture.
