# Supplementary material for: Overexpression of SEPALLATA3-like Gene SnMADS37 Generates Green Petal-Tip Flowers in Solanum nigrum
Source: Plants (Basel). 2025 Jun 20;14(13):1891. doi: 10.3390/plants14131891 (PMC12251700; doi:10.3390/plants14131891)
Supplement: Supplementary file 1 [file plants-14-01891-s001.zip › plants-3627425-supplementary.pdf]

Supplementary materials

Overexpression of *SEPALLATA3*-like gene *SnMADS37* generates green petal-tip flowers in *Solanum nigrum*

Siming Yuan †, Chun-Lan Piao †, Xinyu Zhang, Min-Long Cui *

Key Laboratory of Quality and Safety Control for Subtropical Fruit and Vegetable, Ministry of Agriculture and Rural Affairs, Collaborative Innovation Center for Efficient and Green Production of Agriculture in Mountainous Areas of Zhejiang Province, College of Horticulture Science, Zhejiang A&F University, Hangzhou 311300, China.

† These authors contributed equally to this work.

* **Correspondence:** Min-Long Cui,

E-mail: minlong.cui@zafu.edu.cn

Supplemental Table S1: The primers used for *SnMADS37* gene cloning, detection of transformed plants and RT-PCR analysis in this study.

BamH I-SnMADS37F:	AA**GGATCC**ATGGGAAGGGGTAGGGTTGA
Sac I-SnMADS37R:	AA**GAGCTC**TCAAGGCAACCAGCCAGCCA
SnMADS37-F2:	ATGGGAAGGGGTAGGGTTGA
SnMADS37-R2:	TCAAGGCAACCAGCCAGCCA
SnGLO-F2:	ATGGGGAGAGGAAAGATAGA
SnGLO-R2:	TTAGAACCTTTGGTGCAAATT
SnDEF-F2:	ATGGGTCGTGGAAAAATCGAA
SnDEF-R2:	TCAGGAGAGACATAGATCACG
35sP-F:	GTGTGGGTCAATAATCAGGAAG
NPT II-F:	AGATGGATTGCACGCAGGTTC
NPT II-R:	GTGGTCGAATGGGCAGGTAG
SnChlH-F:	ACTCCCTGAGGAAGCAGTAGA
SnChlH-R:	GCTAGCAGCCACACCAATGAT
SnCLH-F:	ATGCACATAGCCAAGCAGATG
SnCLH-R:	CGAGTCTGGGTATGCCATTG
SnPPH-F:	TGGTCCTAGGTCTGCTGAAGA
SnPPH-R:	CCGTGCACAAATCAAGAGACA
SnActin-F:	CGCGCGCTACACTGTATTCAA
SnActin-R:	TACAAAGGGCAGGGACGTAGTCAA
VIGS-F:	AAGGTACCCAGAGAAAGGAACATGCATTG
VIGS-R:	AATCTAGAAAGGCAACCAGCCAAGCCAT

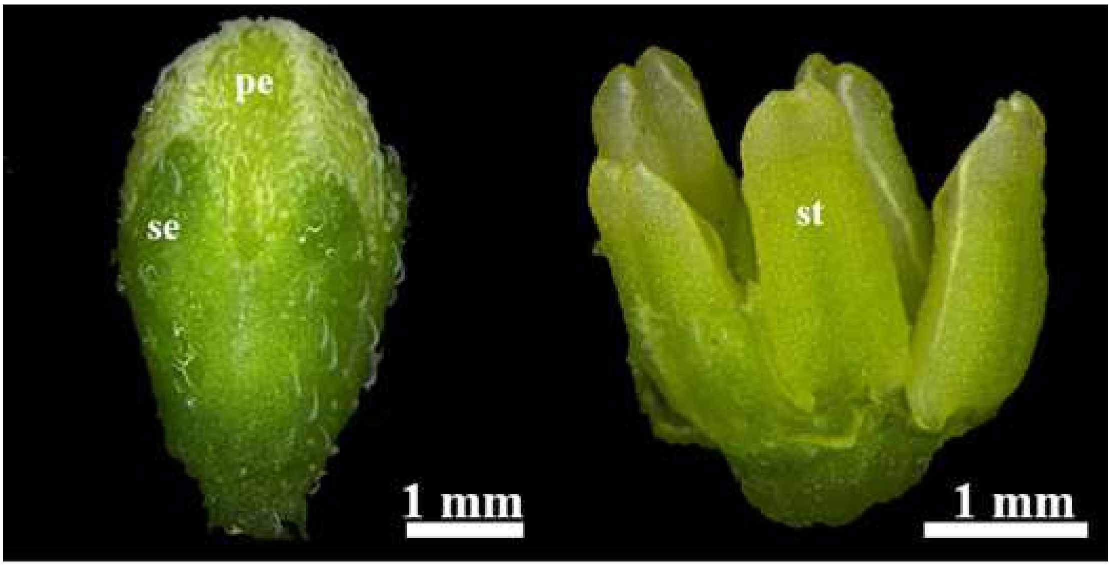

Supplemental Figure S1: The green color of early wild type stage 1 floral bud (A), and with stamens (B) that removed the sepals and petals in Figure (4A). se: sepal, pe: petal, st: stamen.

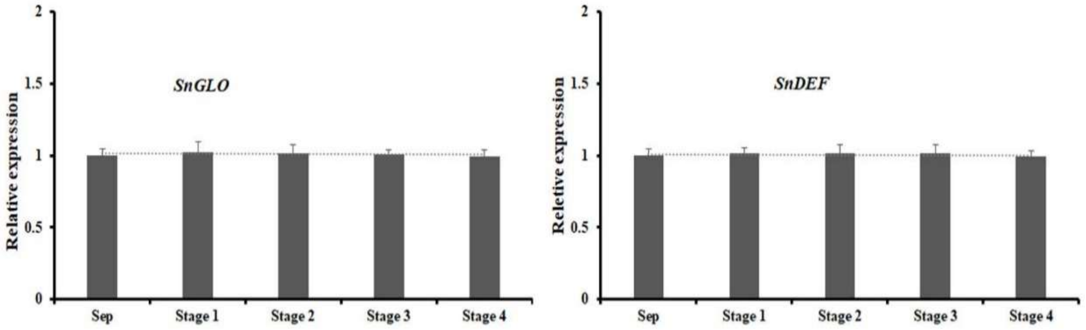

Supplemental Figure S2: Expression analysis of B-function gene in petal development stage of WT. The B-function gene *SnGLO* and *SnDEF* expression were detected in four petal development stages of WT (Fig. 4A). The error bar is indicated as the mean±SD of three biological replicates.

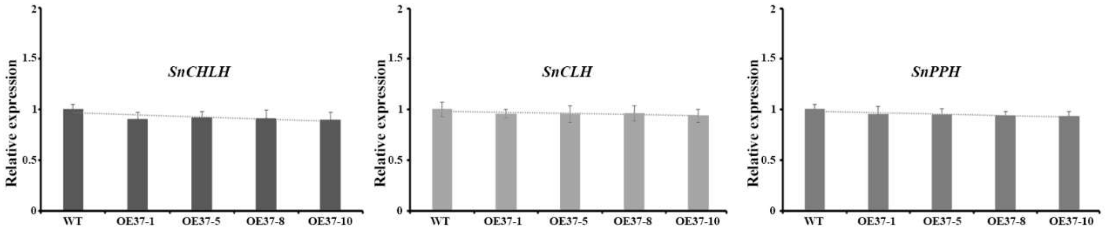

Supplemental Figure S3: Relative expression analysis of chlorophyll-related genes in fully opened stage 4 petals among a WT and four transgenic *S. nigrum* plants. (A) Expression of a putative Mg-chelatase subunit H gene (*SnCHLH*) related to chlorophyll biosynthesis; (B) Expression of a putative hydroxymethyl chlorophyll a reductase gene (*SnCLH*) related to chlorophyll degradation; (C) Expression of a putative pheophytinase gene (*SnPPH*) related to chlorophyll degradation. OE37-1, 5, 8 and 10: Four transformed plants with pBI-*35S::SnMADS37*. Asterisks indicate statistically significant differences between the WT and transgenic lines (n = 3; *p < 0.05; **p < 0.01).

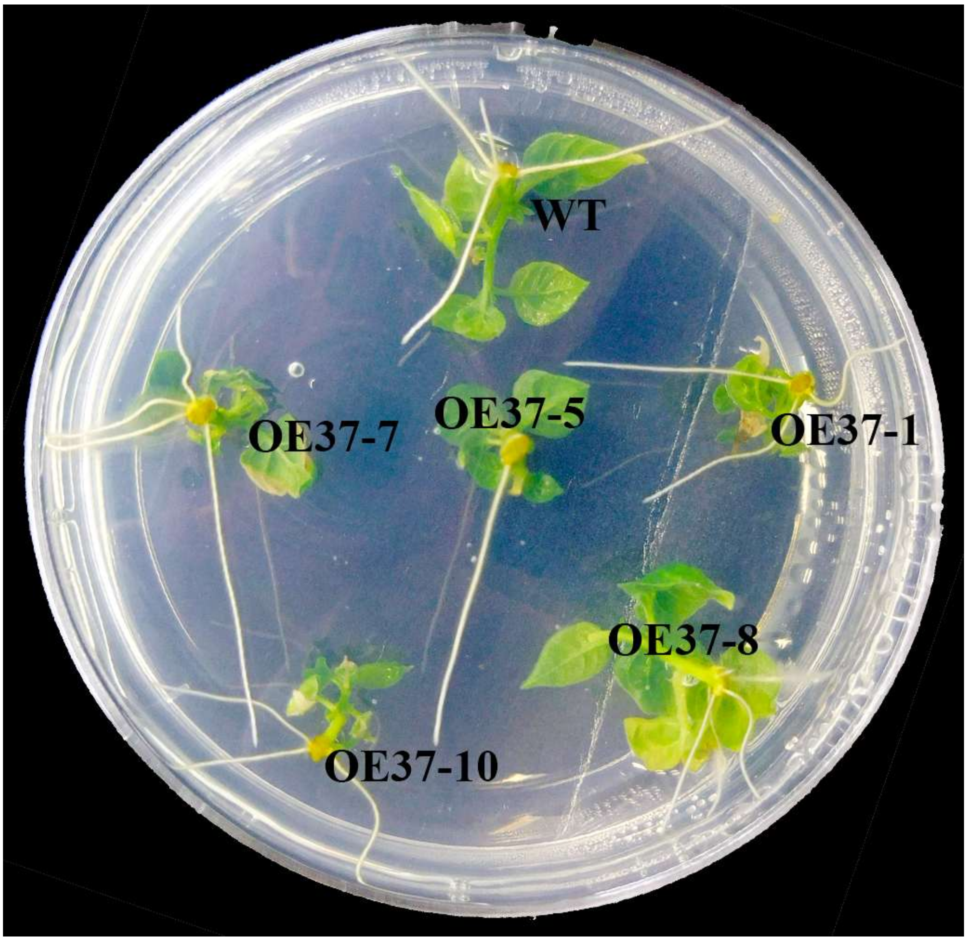

Supplemental Figure S4： Comparison of root coloration between a WT and five overexpressed SnMASD37 plants on MS medium + 200mg /L cefotaxime after three weeks culture. WT: wild type plant; OE37-1, 5, 7, 8, and -10: five independent plants of transformed with 35S::SnMADS37.
